# Examine the effects of Atomoxtine alone versus Atomoxtine combined with Risperidone at sample of Egyptian children suffering from ADHD

**DOI:** 10.1192/j.eurpsy.2025.2077

**Published:** 2025-08-26

**Authors:** M. M. Hamouda

**Affiliations:** 1 Psychiatry , Al-Azhar Faculty Of Medicine , Cairo, Egypt

## Abstract

**Introduction:**

A frequent developmental condition of inattention that may or may not be accompanied by hyperactivity is known as Attention Deficit Hyperactivity Disorder.^1^One’s inability to focus, excessive activity, and behavior that is inappropriate for their age, all of these traits are indicative of ADHD.^2^The data for Atomoxetine indicates that it is safe and effective in treating ADHD in children and teenagers^3^. For kids and teenagers with disruptive behaviors, ADHD, and developmental disorders, Risperidone may be a safe, effective medication. Numerous investigations have demonstrated the high effectiveness of risperidone^4^

**Objectives:**

To study the Efficacy of Atomoxetine alone vs. Atomoxtine with Antipsychotic (Risperidone) on ADHD children.

**Methods:**

This follow-up study was carried out from January to June 2024 on 54 ADHD children (6–18 years old) who were receiving treatment at the psychiatry clinic at Al Hussian University Hospital in Cairo, Egypt. This study was authorized by the Al-Azhar Faculty of Medicine’s Ethical Committee. Following an explanation of the purpose of the study and the acquisition of verbal agreement, all children underwent semi-structured clinical interviews and were excluded from other psychiatric & medical conditions.

Based on the DSM IV criteria, ADHD has been diagnosed in all of the study children. Conner’s 2 test for ADHD, or SCID, was used for every child in the study. Implementing medicinal treatment for every child and monitoring their progress, who were divided into two groups. The first group, which consisted of 27 children diagnosed as ADHD children, received only Atomoxetine, independent of the kind of ADHD. In contrast, 27 recently diagnosed children with ADHD, irrespective of their kind of ADHD, received a combination of Atomoxetine and antipsychotic(Risperidone) medication in the second group.

SPSS 20.0 was employed. The qualitative data were expressed in terms of percentages and figures, and the significance of the outcomes was assessed at the 5% level.

**Results:**

When Atomoxetine was administered to 27 children, it demonstrated a moderate level of efficacy to 12 from 27 patients (44.4%) for all children with ADHD, regardless of type, but it significantly improved the inattention type of ADHD in 4 from 4 patients (100%).Given to 27 children, the combination of Atomoxetine and Risperidone demonstrated greater effect on 24 from 27 patients with (88.9%) for all ADHD children, and it showed a discernible improvement on 9 out of 9 with (100%) for ADHD hyperactivity type.

**Conclusions:**

All types of ADHD are responsive to Atomoxetine or Atomoxetine / Antipsychotic (Risperidone) combination, But Atomoxetine effect is more on Attention deficit variant than other variants, while Antipsychotic (Risperidone) is more effective on hyperactivity and aggression.

**Disclosure of Interest:**

None Declared

